# First Molecular Identification of Canine Parvovirus Type 2 (CPV2) in Chile Reveals High Occurrence of CPV2c Antigenic Variant

**DOI:** 10.3389/fvets.2020.00194

**Published:** 2020-05-05

**Authors:** Cristobal Castillo, Victor Neira, Pamela Aniñir, Sofia Grecco, Ruben Pérez, Yanina Panzera, Nhur-Aischa Zegpi, Alberto Sandoval, Daniel Sandoval, Sergio Cofre, Rene Ortega

**Affiliations:** ^1^Departamento de Patología y Medicina Preventiva, Facultad de Ciencias Veterinarias, Universidad de Concepción, Chillán, Chile; ^2^Departamento de Medicina Preventiva Animal, Facultad de Ciencias Veterinarias y Pecuarias, Universidad de Chile, Santiago, Chile; ^3^Sección Genética Evolutiva, Departamento de Biología Animal, Instituto de Biología, Facultad de Ciencias, Universidad de la República, Montevideo, Uruguay; ^4^Departamento de Ciencias Clínicas, Facultad de Ciencias Veterinarias Universidad de Concepción, Chillán, Chile

**Keywords:** canine parvovirus type 2, CPV2c, Chile, genetic characterization, South America

## Abstract

Canine parvovirus type 2 (CPV2) is one of the most important intestinal pathogens in dogs and puppies. CPV2 has been evolved into three genetic and antigenic variants (2a, 2b, and 2c), which are distributed worldwide. We reported the first study of genetic diversity of CPV2 in Chile. Sixty-five samples were collected from puppies presenting with severe gastroenteritis and different vaccination statuses. PCR, restriction fragment length polymorphism (RFLP), and partial sequencing of the coding region of the structural viral protein VP2 was performed. Thirty of a total of 65 samples tested positive by PCR out of which 19 were further classified as CPV2c and one as CPV2a using RFLP and Sanger sequencing. The phylogeny was in concordance with the RFLP analysis. This is the first report of the genetic characterization of CPV2 in Chile and reveals a high occurrence of CPV2c.

## Introduction

Canine parvovirus (CPV) is a non-enveloped, linear, single-stranded DNA virus that belongs to the family *Parvoviridae*, genus *Protoparvovirus*. The CPV2 genome is 5.2 kb long containing two open reading frames (ORFs) (1). The non-structural ORF encodes two non-structural proteins (NS1 and NS2), the structural ORF encodes the capsid proteins VP1 and VP2 (2). The original CPV strain designated as type-2 (CPV2) was reported in the 1970s and soon after that in the 1980s, two antigenic variants termed CPV types 2a (CPV2a) and 2b (CPV2b) were reported (3). Today, the antigenic variants of CPV2 are classified based on the amino acid present at position 426 of the VP2 capsid protein, asparagine (Asn) for CVP2a, aspartic acid (Asp) for CPV2b, and glutamic acid (Glu) for CPV2c (3). The CVP2a was the first antigenic variant identified and is still ubiquitous. The CVP2b was first reported in 1984 in the United States and is now detected worldwide (4). The CVP2c was first identified in the 2000s in Italy (3) and later reported in Europe, Asia, and South America (5). Most recently, new antigenic variants of CPV2a/b emerged, generated by a specific mutation at position 297 (Ser-Ala), which has been denominated as new CPV2a/b variants (6–9).

In Chile, serologic evidence of CPV2 has been reported in both domestic dogs and wild canids (10, 11). CPV2 has previously been described in Chile; however, information about the genetic and antigenic diversity is not known. The aim of this study was to determine the genetic diversity of CPV2 in Chile.

## Materials and Methods

Small-animal veterinarians were recruited to participate in sample collection during 2016–2017. Fecal samples from diarrhea cases suspected for CPV were collected with detailed clinical history and stored properly at −20°C until used. Data about age, gender, and vaccination status were recorded for each sample. DNA was extracted by fast boiling preparation protocol (12). Briefly, 100 μl of diarrheic fecal material was homogenized in 1 ml of phosphate buffered saline and boiled for 10 min. The preparation was centrifuged at 1,600G for 5 min, and the supernatant containing the genetic material was collected and stored at −20°C for further use. For CPV2 diagnosis, a fragment of VP2 (583 bp) was amplified following the protocol described by Buonavoglia et al. (13). The CPV-positive samples were further processed for another PCR, restriction fragment length polymorphism (RFLP) analysis, and Sanger sequencing. Briefly, a fragment of VP2 (1,315 bp) was amplified by PCR using primers forward 5′-GGA AAC CAA CCA TAC CAA CTC C-3′ and reverse 5′-GGA TTC CAA GTA TGA GAG GC-3′. The RFLP was performed with the restriction enzyme *MboII* for 2 h at 37°C. Digested products (10 μl) were run on a 0.8% agarose gel to see the RFLP pattern. All positive samples were submitted for Sanger sequencing. The sequences were aligned and trimmed in Clustal W using the MEGA 7.0 analysis software (14). The phylogenetic tree was constructed using CPV2 reference sequences including those reported from neighboring countries. The phylogenetic tree was generated by the Bayesian method using MrBayes 3.2 software (15, 16) running 2 million iterations, sampling every 50 iterations, with the first 25% of samples discarded as burn-in. The phylogenetic tree was visualized and edited with FigTree (17).

## Results and Discussion

Sixty-five clinical cases compatible with CPV2 infection were collected from three different locations in Chile, separated by distances ranging from 70 to 470 km. All samples were obtained from puppies younger than 7 months of age, which presented with severe diarrhea and other symptoms such as fever, vomiting, anorexia, and dehydration. Data about vaccination status, gender, and age are described in Supplementary Table 1.

The 46% (30 out of 65) of the samples that tested positive by PCR indicate the important role of CPV2 in causing gastroenteritis in puppies. In general, positive animals were unvaccinated or had an incomplete vaccination schedule, reinforcing the need to increase vaccination efforts to reduce CPV2 prevalence. However, the results suggested that there may be other causative agents associated with severe diarrhea that require further investigation. RFLP was performed successfully on 20 samples only, and the remaining 10 samples which were weak positive by PCR could not be characterized. The RFLP analysis classified 19 samples as CPV2c and one as CPV2a (Supplementary Figure 1). CPV2b variant was not identified.

**Figure 1:**
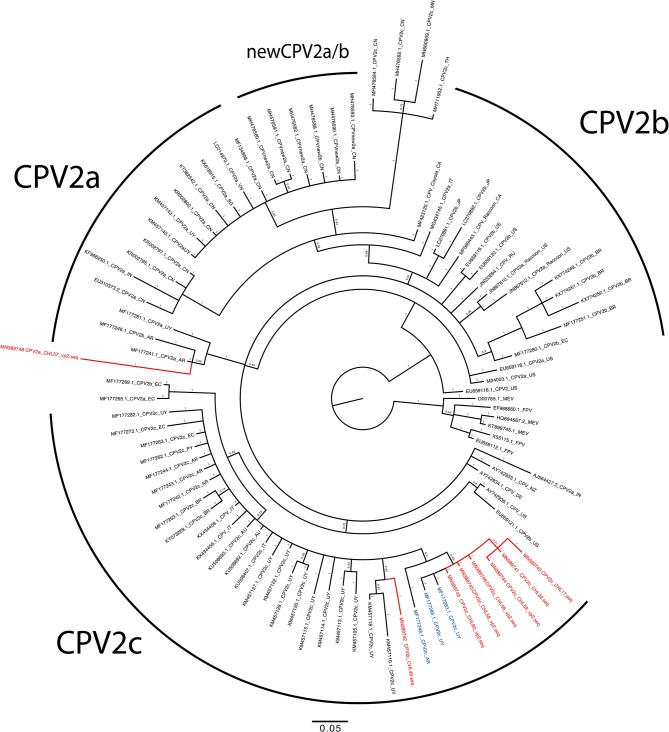
Phylogenetic tree of partial genome of VP2 gene of canine parvovirus (CPV) from Chile, South American strains, and references strains. The phylogenetic tree was constructed by using the Bayesian method using Mr. Bayes running 2 million iterations, sampling every 50 iterations, with the first 25% samples discarded as burn-in. The posterior probability values are indicated in each node. Chilean sequences are highlighted in red, and relevant sequences from Argentina and Uruguay are depicted in blue.

Sanger sequencing was attempted for 30 PCR positive samples; however, only 13 were successfully sequenced and deposited in GenBank (accession numbers MN389736–MN389748). The alignment analysis confirmed the identify of CPV2 variants in concordance with the RFLP analysis (Supplementary Table 2 and Supplementary Figure 2). Chilean CPV2c sequences showed high identity between them (99.9%), and NCBI BLAST had 99.7 to 100% pairwise nucleotide identity values when compared with sequences reported in Uruguay, Argentina, and Mexico. Three samples had an Ala440Thr amino acid substitution, which is antigenically relevant because of its external position in the viral capsid (3, 18, 19). The same amino acid substitution has been observed in Argentina (19) and may suggest that Chilean and Argentine CPV2c populations may have the same origin. Finally, the phylogeny was in concordance of RFLP and alignment analysis. All the Chilean CPV2c were grouped with sequences from Uruguay, Argentina, Paraguay, Ecuador, and other countries all antigenically classified as CVP2c (Figure 1). On the other hand, the closest sequence to the CPV2a strain using nucleotide BLAST was EC/01/2017 (MG264075.1) with 100% identity. The CVP2a strain was identified as a singleton which is genetically related with both CPV2a and CPV2b sequences.

This is the first study describing the genetic diversity of CPV2 in Chile. Previous studies in Chile have reported the serologic identification of CPV2 in both domestic and wild canids, demonstrating that the virus is ubiquitous in Chile. However, genetic variants of CPV2 have been reported from different countries in South America, indicating diversity of this virus (5, 20–22). This study highlighted the high occurrence of CPV2c antigenic variant in puppies affected with severe diarrhea from Chillan city and its widespread presence in three distant locations (470 km) (Supplementary Figure 3). CPV2c variant is considered an emerging pathogen causing epizootics in several countries around the world (3, 23, 24). The occurrence of CPV2c variant has been described in other South American countries including Uruguay, Argentina (19, 25), and Brazil (7, 26, 27). Recently, it has been reported that the CPV2c variants from Argentina originated from Europe and later spread to Brazil and Uruguay (5) and, based on our results, also probably to Chile. On the other hand, CPV2a is still circulating in domestic dogs in South America and predominant in Peru (21), Colombia (22), and recently an increase in the number of cases of this strain has been reported in Uruguay (25). The most recent reports from Latin America demonstrate the presence of the new CPV2a in Ecuador (28), Columbia (22), Brazil (7), and the Caribbean (29).

The small sample size from three cities is a main limitation of this study. Hence, the results may not be representative of the genetic and antigenic diversity of CPV2 in the whole country. However, our results confirm the presence of two variants, CPV2a and CPV2c, in Chile. The CPV2c variant is the predominant variant in Chillan city, but the low number of samples in Los Andes and Santiago may not represent the real diversity. In spite of these limitations, the results confirm that CVP2c is widespread in Chile in a range of 470 km. Further studies are required to confirm the predominance of CPV2c across the country as well as to confirm the absence of CPV2b, which was not identified in this study. We only analyzed partial genomes, but in general, all studies target the VP2 because it is the major antigenic protein.

In conclusion, this is the first genetic characterization of the CPV2 in Chilean dog populations the results of which confirm the widespread occurrence of the CPV2c variant. Future investigations particularly molecular epidemiological studies should be conducted to understand the impact of this virus on domestic and wild canine populations.

## Data Availability Statement

The data for this manuscript is uploaded to the NCBI GenBank (accession numbers MN389736–MN389748).

## Ethics Statement

The animal study was reviewed and approved by Comité de Bioetica de la Facultad de Ciencias Veterinarias, Universidad de Concepcion. Written informed consent for participation was not obtained from the owners because the animals were not identified individually.

## Author Contributions

CC, VN, RO, SG, and RP wrote the manuscript. VN, PA, and SC collected samples. PA, N-AZ, AS, VN, SG, RP, YP, DS, and RO performed the data analysis and data interpretation. RP and RO were in charge of the study design.

## Conflict of Interest

The authors declare that the research was conducted in the absence of any commercial or financial relationships that could be construed as a potential conflict of interest.
