# Supplementary material for: First Molecular Identification of Canine Parvovirus Type 2 (CPV2) in Chile Reveals High Occurrence of CPV2c Antigenic Variant
Source: Front Vet Sci. 2020 May 5;7:194. doi: 10.3389/fvets.2020.00194 (PMC7216333; doi:10.3389/fvets.2020.00194)
Supplement: Supplementary file 1 [file Data_Sheet_1.docx]

Supplementary Material

**Supplementary Table 1.** Sample identification for clinical cases including in study. Identification of gender, age, vaccination status, city of origin, PCR results and RFLP characterization were included.

| N° | Sex | Age  (months) | Vaccination  status | PCR | RFLP | City |
| --- | --- | --- | --- | --- | --- | --- |
| 1 | F | 4 | 1 | - | NT | Chl |
| 2 | F | 4 | 1 | - | NT | Chl |
| 3 | M | 4 | - | - | NT | Chl |
| 4 | M | 2 | - | + | CPV2c | Chl |
| 5 | M | 3 | 2 | - | NT | Chl |
| 6 | M | 5 | - | - | NT | Chl |
| 7 | F | NA | - | - | NT | Chl |
| 8 | M | 3 | - | + | NT | Chl |
| 9 | M | 4 | - | + | CPV2c | Chl |
| 10 | NA | 1 | - | - | NT | Chl |
| 11 | M | 5 | - | + | NT | Chl |
| 12 | F | 2 | 1 | - | NT | Chl |
| 13 | M | 3 | - | - | NT | Chl |
| 14 | NA | NA | - | - | NT | Chl |
| 15 | M | 7 | - | - | NT | Chl |
| 16 | M | 4 | - | - | NT | Chl |
| 17 | NA | NA | - | + | CPV2c | Chl |
| 18 | M | 3 | - | + | NT | Chl |
| 19 | M | 4 | - | + | CPV2c | Chl |
| 20 | F | 3 | - | - | NT | Chl |
| 21 | M | 4 | - | - | NT | Chl |
| 22 | M | 1 | - | - | NT | Chl |
| 23 | M | 3 | - | + | NT | Chl |
| 24 | F | 7 | - | - | NT | Chl |
| 25 | M | 2 | 1 | + | CPV2c | Chl |
| 26 | NA | NA | - | + | CPV2c | Chl |
| 27 | F | 3 | - | + | NT | Chl |
| 28 | F | 5 | - | + | CPV2c | Chl |
| 29 | NA | NA | - | + | CPV2c | Chl |
| 30 | M | 5 | - | + | NT | Chl |
| 31 | F | 4 | - | - | NT | Chl |
| 32 | M | 1 | - | + | CPV2c | Chl |
| 33 | F | 2 | - | - | NT | Chl |
| 34 | F | 4 | 1 | + | CPV2c | Chl |
| 35 | M | 3 | - | + | NT | Chl |
| 36 | F | 3 | - | + | NT | Chl |
| 37 | M | 3 | - | + | CPV2c | Chl |
| 38 | M | 2 | - | + | NT | Chl |
| 39 | F | 4 | - | + | NT | Chl |
| 40 | F | 3 | - | + | CPV2c | Chl |
| 41 | F | 5 | 1 | - | NT | Chl |
| 42 | M | 5 | 2 | - | NT | Chl |
| 43 | NA | 1 | - | - | NT | Chl |
| 44 | NA | 1 | - | - | NT | Chl |
| 45 | NA | 1 | - | - | NT | Chl |
| 46 | F | 3 | 1 | - | NT | Chl |
| 47 | F | 4 | - | - | NT | Chl |
| 48 | F | 6 | - | - | NT | Chl |
| 49 | M | 3 | - | + | CPV2c | Chl |
| 50 | M | 2 | - | - | NT | Chl |
| 51 | M | 6 | - | - | NT | Chl |
| 52 | M | 6 | - | - | NT | Chl |
| 53 | F | 3 | - | - | NT | Chl |
| 54 | M | 3 | - | - | NT | Chl |
| 55 | F | 5 | 1 | + | CPV2c | Chl |
| 64 | NA | NA | NA | + | CPV2c | Stgo |
| 65 | F | NA | NA | - | NT | LA |
| 66 | NA | NA | NA | - | NT | Stgo |
| 67 | F | NA | NA | + | CPV2a | LA |
| 68 | NA | NA | NA | - | NT | Stgo |
| 69 | M | NA | - | + | CPV2c | LA |
| 70 | M | NA | - | + | CPV2c | Stgo |
| 71 | NA | NA | - | + | CPV2c | Stgo |
| 72 | M | NA | - | - | NT | Stgo |
| 73 | NA | NA | - | + | CPV2c | Stgo |

^F = female; M = male; NA= not available; NT = not tested; Chl= Chillan; Stgo= Santiago; LA=Los Andes^

| **Supplementary Table 2** VP2 nucleotide and amino acid mutations | Mutation sites: amino acid residue. |  |  |  |  |  |  |  |  |  |  |  |  |  |  |  | *N/A: Sequence not avalaible; CPV-2, CPV-2a, CPV-2b, CPV-2c: Reference strain; Accession numbers Genbank New Cpv-2a (Ecu): MG264075; CPV-2a (Arg): MF177241; CPV-2a (Uy): MF177281; CPV-2c (Uy): KM457118; |
| --- | --- | --- | --- | --- | --- | --- | --- | --- | --- | --- | --- | --- | --- | --- | --- | --- | --- |
|  |  | 514  A→S | - | - | - | S | - | - | - | - | - | S | - | - | - | - |  |
|  |  | 440  T→A | A | - | - | A | - | - | - | - | - | - | - | - | - | A |  |
|  |  | 426  E→N | - | - | - | N | N | N | N | - | N | N | - | N | - | - |  |
|  |  | 324  Y→I | - | - | - | I | - | - | - | - | - | I | - | - | - | - |  |
|  |  | 297  A→N(S) | - | - | - | N | S | N | S | - | S | N | N | N | - | - |  |
|  |  | 14  A→T(V) | - | - | T | - | V | - | - | - | - | - | - | - | T | - |  |
|  | Strain |  | CPV2c CHL- 17, 19, 32, 37, 64, 70, 71, 73 | CPV2c CHL-28 | CPV2c CHL-49 | CPV2a CHL-67 | CPV-2 | CPV-2a | CPV-2b | CPV-2c | CPV-Vaccine | New CPV-2a (Ecu) | CPV-2a (Arg) | CPV-2a (Uy) | CPV-2c (Uy) | CPV-2c (Arg) |  |

**Supplementary Figures.**


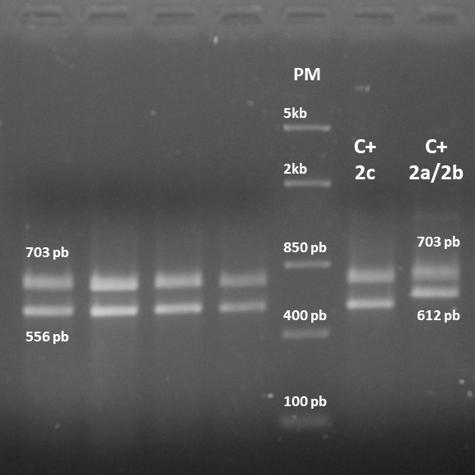


**Supplementary Figure 1.** Electrophoresis agarose gel (0.8%) for CPV2 antigenic classification by RFLP. Lanes 1 to 4 clinical samples classified as CPV2c; Lane molecular weight marker; Lane 5 and 7 CPV2 controls.

*280 290 300 310 320*

*....|....|....|....|....|.*..|....|....|....|....|*

**CPV-2c_CHL9**  **KPCRLTHTWQTNRALGLPPFLNSLPQAEGGTNFGYIGVQQDKRRGVTQMG**

**CPV-2c_CHL17**  **..................................................**

**CPV-2c_CHL19**  **..................................................**

**CPV-2c CHL28**  **..................................................**

**CPV-2c_CHL32** **..................................................**

**CPV-2c_CHL37** **..................................................**

**CPV-2c_CHL49** **..................................................**

**CPV-2c_CHL64** **..................................................**

**CPV-2c_CHL69** **..................................................**

**CPV-2c_CHL70** **..................................................**

**CPV-2c_CHL71** **..................................................**

**CPV-2c_CHL73** **..................................................**

**CPV-2a_CHL67** **..........................N.......................**

*330 340 350 360 370*

*...*|....|....|....|....|....|....|....|....|....|*

**CPV-2c_CHL9**  **NTNYITEATIMRPAEVGYSAPYYSFEASTQGPFKTPIAAGRGGAQTDENQ**

**CPV-2c_CHL17**  **..................................................**

**CPV-2c_CHL19**  **..................................................**

**CPV-2c CHL28**  **..................................................**

**CPV-2c_CHL32** **..................................................**

**CPV-2c_CHL37** **..................................................**

**CPV-2c_CHL49** **..................................................**

**CPV-2c_CHL64** **..................................................**

**CPV-2c_CHL69** **..................................................**

**CPV-2c_CHL70** **..................................................**

**CPV-2c_CHL71** **..................................................**

**CPV-2c_CHL73** **..................................................**

**CPV-2a_CHL67** **...I..............................................**

*380 390 400 410 420*

*....|....|....|....|....|....|....|....|....|....|*

**CPV-2c_CHL9**  **AADGDPRYAFGRQHGQKTTTTGETPERFTYIAHQDTGRYPEGDWIQNINF**

**CPV-2c_CHL17**  **..................................................**

**CPV-2c_CHL19**  **..................................................**

**CPV-2c CHL28**  **..................................................**

**CPV-2c_CHL32** **..................................................**

**CPV-2c_CHL37** **..................................................**

**CPV-2c_CHL49** **..................................................**

**CPV-2c_CHL64** **..................................................**

**CPV-2c_CHL69** **..................................................**

**CPV-2c_CHL70** **..................................................**

**CPV-2c_CHL71** **..................................................**

**CPV-2c_CHL73** **..................................................**

**CPV-2a_CHL67** **..................................................**

*430 440 450 460 470*

*....|....|....|....|....|....|....|....|....|....|*

**CPV-2c_CHL9**  **NLPVTEDNVLLPTDPIGGKAGINYTNIFNTYGPLTALNNVPPVYPNGQIW**

**CPV-2c_CHL17**  **..................................................**

**CPV-2c_CHL19**  **..................................................**

**CPV-2c CHL28**  **...................T..............................**

**CPV-2c_CHL32** **..................................................**

**CPV-2c_CHL37** **..................................................**

**CPV-2c_CHL49** **...................T..............................**

**CPV-2c_CHL64** **..................................................**

**CPV-2c_CHL69** **..................................................**

**CPV-2c_CHL70** **..................................................**

**CPV-2c_CHL71** **..................................................**

**CPV-2c_CHL73** **..................................................**

**CPV-2a_CHL67** **.....N.............T..............................**

*480 490 500*

*....|*...|....|....|....|....|*

**CPV-2c_CHL9**  **DKEFDTDLKPRLHVNAPFVCQNNCPGQLF***

**CPV-2c_CHL17**  **.............................***

**CPV-2c_CHL19**  **.............................***

**CPV-2c CHL28**  **.............................***

**CPV-2c_CHL32** **.............................***

**CPV-2c_CHL37** **.............................***

**CPV-2c_CHL49** **.............................***

**CPV-2c_CHL64** **.............................***

**CPV-2c_CHL69** **.............................***

**CPV-2c_CHL70** **.............................***

**CPV-2c_CHL71** **.............................***

**CPV-2c_CHL73** **.............................***

**CPV-2a_CHL67** **.............................***

**Supplementary Figure 2.** Partial alignment (228aa) of VP2 capsid protein of 13 Chilean CPV2 strains. The box shows four mutation at 297, 324, 440 residues. The 426 residue classified CPV2a strains (asparagine, N) and CPV2c (glutamate, E).


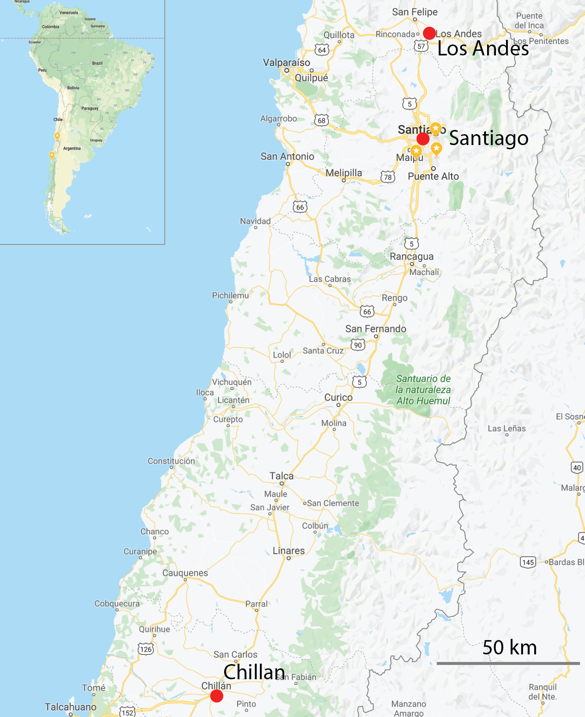


**Supplementary Figure 3.** Map of Chile indicating cities including in the study (red circles).
